# The use of a proactive dissemination strategy to optimize reach of an internet-delivered computer tailored lifestyle intervention

**DOI:** 10.1186/1471-2458-13-721

**Published:** 2013-08-05

**Authors:** Francine Schneider, Daniela N Schulz, Loes HL Pouwels, Hein de Vries, Liesbeth ADM van Osch

**Affiliations:** 1 CAPHRI / Department of Health Promotion, Faculty of Health, Medicine and Life Sciences, Maastricht University, Maastricht, The Netherlands; 2 Regional Public Health Service / Southeast Brabant, Helmond, The Netherlands

**Keywords:** Internet-delivered interventions, Online interventions, Reach, Computer tailoring, Lifestyle, User characteristics, Proactive dissemination

## Abstract

**Background:**

The use of reactive strategies to disseminate effective Internet-delivered lifestyle interventions restricts their level of reach within the target population. This stresses the need to invest in proactive strategies to offer these interventions to the target population. The present study used a proactive strategy to increase reach of an Internet-delivered multi component computer tailored intervention, by embedding the intervention in an existing online health monitoring system of the Regional Public Health Services in the Netherlands.

**Methods:**

The research population consisted of Dutch adults who were invited to participate in the Adult Health Monitor (N = 96,388) offered by the Regional Public Health Services. This Monitor consisted of an online or a written questionnaire. A prospective design was used to determine levels of reach, by focusing on actual participation in the lifestyle intervention. Furthermore, adequacy of reach among the target group was assessed by composing detailed profiles of intervention users. Participants’ characteristics, like demographics, behavioral and mental health status and quality of life, were included in the model as predictors.

**Results:**

A total of 41,155 (43%) people participated in the Adult Health Monitor, of which 41% (n = 16,940) filled out the online version. More than half of the online participants indicated their interest (n = 9169; 54%) in the computer tailored intervention and 5168 participants (31%) actually participated in the Internet-delivered computer tailored intervention. Males, older respondents and individuals with a higher educational degree were significantly more likely to participate in the intervention. Furthermore, results indicated that especially participants with a relatively healthier lifestyle and a healthy BMI were likely to participate.

**Conclusions:**

With one out of three online Adult Health Monitor participants actually participating in the computer tailored lifestyle intervention, the employed proactive dissemination strategy succeeded in ensuring relatively high levels of reach. Reach among at-risk individuals (e.g. low socioeconomic status and unhealthy lifestyle) was modest. It is therefore essential to further optimize reach by putting additional effort into increasing interest in the lifestyle intervention among at-risk individuals and to encourage them to actually use the intervention.

**Trial registration:**

Dutch Trial Register (NTR1786) and Medical Ethics Committee of Maastricht University and the University Hospital Maastricht (NL2723506809/MEC0903016).

## Background

Lifestyle behaviors, such as smoking, unhealthy eating habits, physical inactivity and excessive alcohol consumption are established risk factors for chronic diseases, most importantly for cardiovascular diseases, different types of cancer and diabetes type II [1,2]. The high prevalence of these behaviors [2] and their tendency to co-occur [3,4], stresses the need to invest in effective lifestyle interventions.

Currently, lifestyle interventions are more and more frequently disseminated through the Internet. Due to the high level of reach and accessibility of the Internet [5], it offers a good platform for the dissemination of tailored and targeted interventions to the general public [5-8]. Especially interventions using computer tailored techniques have reported positive effects [7-9], e.g. in the fields of physical activity [10,11], fruit and vegetable intake [12,13], smoking [14,15] and alcohol consumption [16,17]. This potentially high level of reach, combined with the proven effectiveness of computer tailored interventions, would suggest that interventions offered through the Internet hold the promise to significantly contribute to an increased impact on public health [18].

However, despite the promising prospects of the Internet, actual reach of effective interventions within the target population remains inadequate [19-22], as only a limited proportion of people is actually reached by the interventions [21,23]. These suboptimal levels of reach might be partially explained by the fact that most effective interventions are offered reactively to the target population [24]. The use of reactive dissemination strategies implies that a rather passive approach is taken in which users themselves must undertake action in order to use and optimally benefit from the intervention content [25]. Potential users must actively look and register for existing lifestyle initiatives. However, several barriers prevent large proportions of the population from actively seeking assistance in changing their lifestyle behaviors. The barriers pertain to insufficient knowledge on the existence of interventions or places where they can be found, unawareness of the current status of their lifestyle behaviors or a lack of motivation to change their lifestyle [26,27]. This stresses the necessity to invest in strategies through which lifestyle interventions can be proactively offered to the target population. These strategies have the potential to diminish important barriers for use and increase the knowledge of existing, evidence-based interventions [28,29].

Within the present study, a proactive strategy was used to increase reach of an Internet-delivered multi component computer tailored intervention, focusing on physical activity, fruit and vegetable consumption, alcohol intake and smoking cessation. In order to proactively offer the intervention to the target population, the intervention was embedded in a nationwide survey, the Adult Health Monitor, of two of the Regional Public Health Services (RPHS) in the Netherlands [30]. As part of their statutory obligation to periodically monitor the health of the Dutch adult population, this survey is used to assess overall levels of health, as well as different aspects of general health, e.g. physical, mental and social health among a representative sample with an interval of four years. Based on findings of this monitoring tool, future health policies at a national, provincial and local level are outlined. Integrating the Internet-delivered computer tailored intervention in the Adult Health Monitor was expected to have several benefits. Most importantly, the Adult Health Monitor was expected to provide an important access point for reaching a considerably large segment of the Dutch population with an evidence-based intervention. Furthermore, by integrating the computer tailored intervention in the Adult Health Monitor, awareness of the existence of the intervention could be increased. Thirdly, by embedding the intervention in the Adult Health Monitor, data can directly be obtained and presented in order to inform people on the status of their current lifestyle. This may increase the level of lifestyle awareness and serve as an important cue to action to positively change their current lifestyle. Finally, integrating an Internet-delivered computer tailored intervention will enable the RPHS’s to optimize their public health education task; besides solely monitoring health behavior, the RPHS’s now have an opportunity to provide people with personalized advice on how to effectively change their lifestyle status.

The main aim of this study was to determine the effect of this proactive dissemination strategy on reach of the Internet-delivered computer tailored intervention. Within the RE-AIM framework, reach is defined as the percentage of people invited and eligible for participation that actually participate in the intervention [18]. Therefore, success of this proactive dissemination strategy was assessed by studying actual levels of participation in the computer tailored lifestyle intervention among people included in the Adult Health Monitor sample. Furthermore, the second aim of this study was to determine adequacy of reach among the target population [28] by composing and analyzing detailed profiles of intervention users [18,31].

## Methods

Within the current study the level of reach of an Internet-delivered computer tailored lifestyle intervention offered through the Adult Health Monitor was examined. Furthermore, adequacy of reach among the target group was assessed by composing detailed profiles of computer tailored intervention users. This study was approved by the Medical Ethics Committee of Maastricht University and the University Hospital Maastricht (NL27235.068.09/MEC 09-3-016) [32,33]. Data for the present study were collected from October 2009 until July 2010.

### Participants and inclusion criteria

The research population consisted of adults (19–64 years) living in the Dutch Provinces of Zeeland and North-Brabant who participated in the Adult Health Monitor 2009. Additional inclusion criteria for participation in the computer tailored intervention were that participants had to be able to understand the Dutch language sufficiently, had to have access to the Internet and had to have a valid e-mail address. There were no explicit exclusion criteria stated for the current study. Participants had to consent to their participation by filling out an online informed consent form.

### Recruitment and procedure

In the fall of 2009, four RPHS’s of the provinces North-Brabant and Zeeland invited a representative sample of inhabitants of these provinces to participate in the Monitor 2009. A total of 96,388 participants received an invitation to participate by postal mail. This invitation included information on the content and purpose of the Adult Health Monitor. Furthermore, a return envelope was included to return the questionnaire after completion. Besides filling out the written Adult Health Monitor questionnaire, participants were also offered an opportunity to fill out an online version of the questionnaire. For this purpose, a link to the website was included in the invitation, as well as personal log-in information. Since the Internet-delivered computer tailored lifestyle intervention was a web-based intervention, it was only available for participants that decided to fill out the online version of the monitor questionnaire. Online participation was therefore encouraged by informing people about the embedded intervention in the invitation letter and by pointing out its exclusive availability for online participants.

After completing the Adult Health Monitor, all participants of the online version were introduced to the embedded computer tailored lifestyle intervention. Participants were explained that the intervention was free of charge and provided an opportunity to receive personalized computer tailored feedback about their health behaviors. Participants indicating to be interested in the intervention were asked to leave their e-mail address and to fill out an online form indicating their consent to participate in the current study. Approximately three weeks after completion of the Adult Health Monitor, all interested participants received an e-mail invitation to log in to the computer tailored intervention with a personal login code and password. After approximately one month, non-responders received an additional e-mail reminding them about their interest in the intervention and inviting them to participate. After logging on to the intervention, participants received detailed information on its goal and content. Data on demographic and behavioral characteristics obtained through the Adult Health Monitor were transported to the computer tailored intervention.

## Materials

The computer tailored intervention integrated interventions that have been tested and proven to be effective in randomized control trials for increasing smoking cessation, promoting the intake of fruit and vegetables, increasing the level of physical activity and reducing the level of alcohol consumption [16,34-37]. The intervention used a dual approach to guide people towards behavior change. The first part consisted of a health risk appraisal and compared participants’ health behavior status to the Dutch public health guidelines set for these behaviors (i.e. being moderately physically active for 30 minutes at least five days a week [38], eating two pieces of fruit per day and eating 200 grams of vegetables per day [39], not drinking more than one (women) or two (men) glasses of alcohol a day [40] and non-smoking) [41]. We used feedback messages to inform people on the current status of each health behavior. All messages were complemented by using graphic representations of traffic lights [42], with a green light corresponding to adherence to the guidelines and a red light corresponding to non-adherence. An orange light was used for people that were close to adherence to these guidelines. In case of discrepancies between current behavior and the guidelines, people were alerted and directed to the computer tailored modules [33].

Second, participants were provided with assistance in changing their health behavior by means of five separate computer tailored modules on physical activity, fruit and vegetable intake, alcohol consumption and smoking cessation. The content of these modules was based on the Integrated Model for exploring motivational and behavioral change (I-Change Model) [26] and used a fixed, gradual approach to guide people towards behavior change. Within the modules, all health advices were adapted to individuals’ demographic, behavioral and cognitive characteristics [43,44]. Demographic and behavioral characteristics, like participants’ gender and current health behavior status were directly obtained through the Adult Health Monitor. Cognitive variables, such as attitude, perceived social influence, self-efficacy, intention and action planning strategies (preparatory plans and coping plans) were assessed by using an additional tailoring questionnaire. An extensive description of the intervention content is described elsewhere [32,33].

### Measures

User characteristics were collected in order to produce detailed profiles of people who decided to participate in the computer tailored intervention in order to determine adequacy of reach. These profiles entailed information on demographic characteristics, health behavior status, mental health status and quality of life, obtained through the Adult Health Monitor.

#### Demographic characteristics

Demographic characteristics included questions on age, gender (1 = male; 2 = female), height, weight, marital status (widower, divorced, unmarried (not in a relationship = 1); or living together, married (in a relationship = 2)), native country (1 = not from the Netherlands; 2 = from the Netherlands), educational level (no education, primary or lower vocational school (low = 1); secondary vocational school or high school (medium = 2); or higher professional education or university (high = 3)) and current work status (unemployed = 1; employed = 2). Data on participants’ height (in meters) and weight (in kilograms) were used to compose a Body Mass Index (BMI). This index was defined by dividing participants’ body weight (kg) by the square of participants’ height (m) and was categorized as <18.5 = 1 (underweight); ≥18.5 to <25 = 2 (normal weigth); ≥25 to <30 = 3 (overweight); ≥30 = 4 (obesity).

#### Health behavior status

Health behavior status was objectively assessed by measuring level of physical activity, fruit and vegetable intake, alcohol consumption and smoking behavior. Physical activity was measured by the Short QUestionnaire to ASsess Health enhancing physical activity (SQUASH) [45]. Fruit and vegetable intake were assessed using the Food-Frequency-Questionnaire (FFQ) for fruit and vegetable intake [46,47]. Alcohol consumption was measured by the Dutch Quantity-Frequency-Variability Questionnaire (QFV) [48] and smoking status was assessed by asking participants whether they smoked, what they smoked (cigarettes, cigars, packets pipe tobacco) and how much they smoked per day (cigarettes) and per week (cigars/ packets pipe tobacco). Furthermore, an additional item was used to obtain a more subjective assessment of participants’ lifestyle status. This item assessed the degree to which participants considered their own current lifestyle to be healthy and was measured on a five point scale (1 = very unhealthy; 5 = very healthy).

#### Mental health status

Mental health status was assessed by using the Kessler Psychological Distress Scale (K10) [49,50]. This scale assesses the occurrence of non-specific psychological distress using ten items measured on a five point scale (1 = none of the time; 5 = all of the time). A sum score was computed, with higher scores indicating higher levels of distress.

#### Quality of life

Quality of life was assessed using the Medical Outcomes Study 12-Item Short Form Health Survey (SF-12) [51,52].

#### Outcome measures

The primary outcome measure was level of actual participation in the pro intervention gram. Secondary outcome measures were medium of participation and level of interest in the intervention. Dichotomous variables were created in order to examine level of participation (0 = no participation/1 = participation), medium of participation (0 = written questionnaire/1 = online questionnaire) and level of interest in the intervention (0 = not interested/1 = interested). Participants of the online Adult Health Monitor were asked to indicate their interest in the intervention by leaving their e-mail address. Interest in the intervention was labeled as “yes” when people filled out their e-mail address. Furthermore, participation was objectively monitored by looking at log-in data and was labeled as “yes” when people responded to the e-mail invitation by logging in to the computer tailored intervention.

### Statistical analysis

First, general descriptive statistics were used to describe user characteristics of the participants, by focusing on demographic characteristics, health behavior status, mental health status and quality of life as well as level of interest and participation in the computer tailored intervention. Second, logistic regression analyses were conducted. Medium of participation, interest and participation in the computer tailored intervention were the dependent variables. Demographic characteristics (age, gender, BMI, marital status, native country, educational level and current work status), health behavior status, mental health status and quality of life were included in the model as predictors. An alpha of .05 was used to indicate statistical significance. All statistical analyses were done with the program SPSS 19.0.

## Results

### Reach of the proactive dissemination strategy

**Figure 1:**
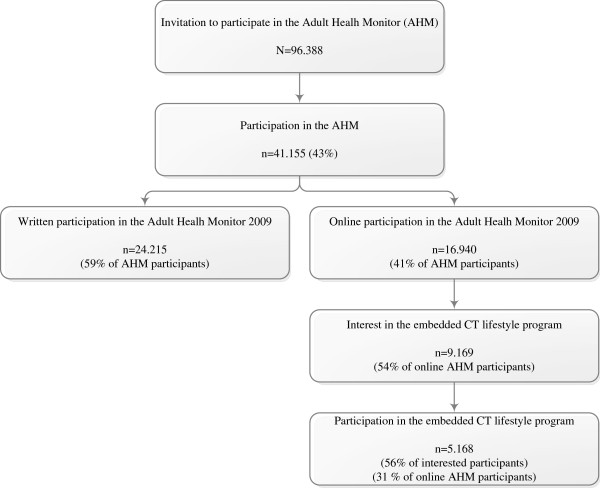
Flowchart of Adult Health Monitor and CT lifestyle intervention participation.

In total, 41,155 (43%) people participated in the Adult Health Monitor 2009 in the provinces of North-Brabant and Zeeland; 24,215 participants (59%) filled out the written version and 16.940 participants (41%) filled out the online version. All online participants were introduced to the Internet-delivered computer tailored lifestyle intervention and 9.169 participants (54%) actually indicated to be interested in the intervention. Finally, all participants that indicated their interest were invited to use the intervention. This resulted in a total of 5168 participants (31% of the online sample and 56% of the sample of people interested in participating) logging in to the Internet-delivered computer tailored intervention (Figure 1).

### Baseline characteristics of intervention users

Users of the Internet-delivered computer tailored intervention had a mean age of 44 years (SD = 12.67), around 54% was male and almost 50% had a medium educational level (Table 1). Furthermore, a little over 75% of all participants was employed and in a relationship and 95% was born in the Netherlands. Approximately 50% of all participants had a healthy body weight, whereas a little less than 2% of all visitors was underweight, 35% was overweight and 10% was obese. With regard to physical activity, 21% did not comply with the Dutch guidelines, whereas 46% and 69% were not adhering to the Dutch guidelines of fruit and vegetables intake respectively. One out of five visitors (19%) indicated that they smoked, whereas approximately one of four participants (28%) did not comply with the Dutch guidelines for alcohol intake. In total, the largest group of people complied with three lifestyle guidelines (36%), whereas a very limited percentage of people complied with none of these guidelines (1%) or all of the guidelines (10%). Characteristics of all Adult Health Monitor participants, online Adult Health Monitor participants and people that indicated their interest in the Internet-delivered computer tailored lifestyle intervention are also listed in Table 1.

**Table 1:** Characteristics of Adult Health Monitor participants (N = 41.155)

	**Total sample N = 41.155**	**Online completion n = 16.940**	**Interest n = 9.169**	**Participation n = 5.168**
Age (19–64) (Mean, SD)	44.78, 12.70	43.19, 12.42	43.23, 12.77	43.94, 12.67
Sex (%)				
*Men*	44.7	51.1	52.6	53.8
*Women*	55.3	48.9	47.4	46.2
Education level (%)				
*Low*	23.2	14.7	13.2	10.6
*Medium*	48.5	48.9	47.7	46.9
*High*	28.2	36.4	39.1	42.6
Work situation (%)				
*Employed*	72.8	77.1	76.5	76.3
*Unemployed*	27.2	22.9	23.5	23.7
Marital status (%)				
*In a relationship*	76.8	77.0	75.4	76.0
*Not in a relationship*	24.2	23.0	24.6	24.0
Native country (%)				
*The Netherlands*	93.3	93.5	93.8	95.0
*Other*	6.7	6.5	6.2	5.0
BMI (kg m^−2^) (%)				
*<18.5*	1.5	1.4	1.5	1.7
*≥18.5 to 25*	50.9	51.5	51.1	52.6
*≥25 to <30*	35.1	35.4	35.6	35.4
*≥30*	12.4	11.7	11.8	10.4
Physical activity (%)				
*Adherence*	78.4	77.1	77.7	79.1
*Non-adherence*	21.6	22.9	22.3	20.9
Vegetable consumption (%)				
*Adherence*	31.2	29.9	30.4	31.5
*Non-adherence*	68.8	70.1	69.6	68.5
Fruit consumption (%)				
*Adherence*	49.7	46.1	45.8	45.9
*Non-adherence*	50.3	53.9	54.2	54.1
Smoking behavior (%)				
*Adherence*	74.7	77.6	78.1	81.7
*Non-adherence*	25.3	22.4	21.9	18.3
Alcohol intake (%)				
*Adherence*	73.6	73.6	72.3	71.7
*Non-adherence*	26.4	26.4	27.7	28.3
Total number of guidelines				
*0*	0.9	0.9	0.9	0.7
*1*	7.2	7.5	7.3	6.5
*2*	21.3	22.1	22.4	20.9
*3*	33.6	34.1	33.9	35.5
*4*	27.1	26.2	26.3	26.6
*5*	9.9	9.1	9.2	9.7
Personal judgment lifestyle				
*Very unhealthy*	5.7	5.5	5.6	6.5
*Unhealthy*	61.8	61.6	60.3	61.8
*Not unhealthy / not healthy*	28.9	28.9	29.4	27.5
*Healthy*	3.2	3.7	4.3	3.9
*Very healthy*	0.4	0.3	0.4	0.3
K10 (Mean, SD)	15.41, 6.26	15.17, 5.90	15.47, 6.08	15.21, 5.71
SF-12 (Mean, SD)	39.76, 5.63	40.09, 5.29	39.88, 5.39	40.12, 5.19

### Adequacy of reach

#### Participation in the online adult health monitor

Results of multiple logistic regression analyses indicated that younger (OR = 0.99; CI 0.99 – 0.99; p < 0.05), male (OR = 0.65; CI 0.62 – 0.68; p < 0.05) and relatively highly educated participants (OR = 2.63; CI 2.45 – 2.81; p < 0.05), as well as participants that were employed (OR = 1.06; CI 1.00 – 1.12; p < 0.05) and in a relationship (OR = 1.09; CI 1.03 – 1.15; p < 0.05) were more inclined to fill out the online version of the Adult Health Monitor. Furthermore, participants with an unhealthier lifestyle (fewer guidelines complied with) were more likely to fill out the online version of the Adult Health Monitor (OR = 0.97; CI 0.95 – 0.99; p < 0.05).

#### Interest in the computer tailored lifestyle intervention

Results of multiple logistic regression analyses indicated that younger (OR = 0.99; CI 0.99 – 0.99; p < 0.05), male (OR = 0.65; CI 0.62 – 0.68; p < 0.05) and relatively highly educated participants (OR = 2.63; CI 2.45 – 2.81; p < 0.05), as well as participants that were employed (OR = 1.06; CI 1.00 – 1.12; p < 0.05) and in a relationship (OR = 1.09; CI 1.03 – 1.15; p < 0.05) were more inclined to fill out the online version of the Adult Health Monitor. Furthermore, participants with an unhealthier lifestyle (fewer guidelines complied with) were more likely to fill out the online version of the Adult Health Monitor (OR = 0.97; CI 0.95 – 0.99; p < 0.05).

#### Use of the computer tailored lifestyle intervention

Results of multiple logistic regression analyses indicated that male (OR = 0.91; CI 0.83 – 0.99; p < 0.05), older (OR = 1.02; CI 1.01 – 1.02; p < 0.05) and relatively highly educated participants (OR = 1.93; CI 1.67 – 2.23; p < 0.05) were more likely to participate in the intervention by logging in to the intervention (see Table 2). Furthermore, intervention participants were more likely to be from the Netherlands (OR = 1.45; CI 1.21 – 1.75; p < 0.05). Finally, participants with a relatively healthier lifestyle (more guidelines complied with) (OR = 1.06; CI 1.01 – 1.10; p < 0.05) and underweighted participants (OR = 1.55; CI 1.06 – 2.27; p < 0.05) were more likely to participate in the computer-tailored intervention, whereas overweighed (OR = 0.88; CI 0.79 – 0.97; p < 0.05) and obese participants (OR = 0.71; CI 0.61 – 0.82; p < 0.05) were less likely to participate.

**Table 2:** Predictors of participation in the CT lifestyle intervention (N = 9.169)

	**CT lifestyle intervention participation**		
	**OR (95% CI)**		
	**OR**	***p***	**CI**
**Demographic characteristics**			
Age	**1.02**	0.000	1.01-1.02
Sex			
*Male (reference)*			
Female	**0.91**	0.033	0.83-0.99
Education			
*Low (reference)*			
*Medium*	**1.54**	0.000	1.34-1.77
*High*	**1.93**	0.000	1.67-2.23
Work situation			
*Unemployed (reference)*			
*Employed*	0.90	0.050	0.80-1.00
Marital status			
*Not in a relationship (reference)*			
*In a relationship*	0.93	0.210	0.83-1.04
Native country			
*Other native country (reference)*			
*The Netherlands*	**1.45**	0.000	1.21-1.75
BMI			
*≥18.5 to 25 (reference)*			
*<18.5*	**1.55**	0.023	1.06-2.27
*≥25 to <30*	**0.88**	0.009	0.79-0.97
*≥30*	**0.71**	0.000	0.61-0.82
**Health behavior status**			
Total # of guideline (adherence)	**1.06**	0.009	1.01-1.10
Personal judgment own current lifestyle	1.06	0.139	0.98-1.15
**Mental health status**			
K10	1.00	0.815	0.99-1.01
**Quality of life**			
SF-12	1.00	0.555	0.99-1.02

Note: Significant OR’s are depicted in bold.

## Discussion

Within the current study, a proactive dissemination strategy was used to optimize reach of an Internet-delivered computer tailored lifestyle intervention. This proactive strategy consisted of embedding the intervention in the Adult Health Monitor of the RPHS’s of two Dutch provinces. Success of this strategy was determined by studying actual levels of participation in the computer tailored lifestyle intervention. Furthermore, we investigated adequacy of reach by studying user characteristics that predicted interest en participation in the computer tailored lifestyle intervention.

### Reach of the proactive dissemination strategy

More than half of the online Adult Health Monitor participants indicated their interest in the intervention. Moreover, more than half of all interested participants actually participated in the computer tailored lifestyle intervention, which is one out of three invited and eligible participants. It is difficult to compare these results to other studies that study reach in a real world setting, since actual estimates of reach are scarce [53]. However, a multi-risk lifestyle behavior intervention offered to a comparable sample reported levels of reach around 3% of the eligible participants [54]. Results from efficacy trials have indicated that actual reach of the interventions is suboptimal [22,54,55], with participation rates ranging from 2.5% to 20%. Considering these results, the current proactive dissemination strategy can be regarded as successful.

### Adequacy of reach

Besides studying actual levels of reach, the current study also aimed at examining adequacy of reach among the target group (i.e. at-risk individuals). This was done by composing profiles of users and by examining which user characteristics predicted both interest and participation in the intervention. Results indicated that especially older, male and relatively highly educated participants were inclined to indicate their interest in the intervention. Furthermore, participants that were unemployed, not in a relationship and born in the Netherlands were more likely to be interested in the computer tailored intervention. Finally, participants with relatively more symptoms of depression and anxiety were more likely to be interested in the computer tailored intervention. Even though the effect of employment status, marital status and depressive symptomatology diminished with regard to actual participation in the intervention, the effect of education and native country remained, with relatively higher educated participants and participants from the Netherlands being more willing to participate in the intervention. This finding is in line with previous studies examining use of Internet-delivered lifestyle interventions, where the majority of participants had a relatively high educational level [54,56-58]. The level of reach was modest in other subgroups, as results indicated that especially participants with a relatively healthier lifestyle and a healthy BMI were likely to actually participate in the intervention. These results are to a large extent in line with previous findings that Internet-delivered computer tailored interventions tend to be predominantly used by people with a healthy lifestyle [43,54,58,59].

### Participation in the adult health monitor

Of the sample that was selected to participate in the Adult Health Monitor 2009, a little over forty percent accepted the invitation to participate. Even though the overall percentages of people that participated in the Monitor has decreased compared to the Adult Health Monitor conducted in 2005 [60], the percentage of people that decided to participate using the online monitoring questionnaire has substantially increased. In 2005, only one out of four respondents preferred to use the online questionnaire [60]. This increase is in line with the rapidly expanding penetration rates of the Internet in the Netherlands. Since 2006, Internet access rates have increased from 65% to almost 93% of all Dutch inhabitants in 2013 [5], allowing for an increased amount of people filling out the online AHM questionnaire.

Participation in the online version of the Adult Health Monitor was preferred by people with an unhealthier lifestyle compared to the written version. These results imply that using the online Adult Health Monitor is a good tool to reach those people that are expected to benefit most from lifestyle interventions (i.e. people that engage in health risk behaviors). However, results on actual participation in the intervention imply that the proactive strategy did not succeed in persuading these at-risk people to actually participate in the embedded computer tailored lifestyle intervention. Even though suboptimal participation rates among people with an unhealthy lifestyle are common for most computer tailored lifestyle interventions [43,54,58,59], the online environment of the Adult Health Monitor offers good opportunities to reach at-risk groups. However, these opportunities might be further optimized in the future. Direct transportation of data from the Adult Health Monitor to the computer tailored lifestyle intervention allowed for composition of an individual health overview addressing all relevant lifestyle behaviors. However, this overview was only presented to participants that were interested in the intervention and decided to actually participate. Impact of this proactive strategy might be further improved if this overview could be presented to all Adult Health Monitor participants, instead of only interested participants. The overview provided personalized information on the status of individuals’ current lifestyle behaviors, as well as additional information regarding the guidelines set for these behaviors. As a result, the detailed content of this overview was very suitable to increase awareness of people’s current lifestyle status and to point out discrepancies with the guidelines set for these behaviors. This overview may then serve as an important cue to action to change their lifestyle among people with an unhealthy lifestyle.

Considering the promising results of the used proactive dissemination strategy with regard to actual levels of reach, the Adult Health Monitor can be regarded as a suitable and promising vehicle to disseminate evidence-based Internet-delivered lifestyle interventions to the general public. The proportion of people reached shows the potential for broad reach when implemented on a national level. Offering effective interventions through the Adult Health Monitor environment at a national level can therefore serve as an important access point to reach the target population. As a consequence, public health impact of these interventions can be further optimized. Even though we partly succeeded in reaching the target population of at-risk individuals, adequacy of reach may be further optimized by putting additional effort into the recruitment of online and at-risk participants. It is therefore recommendable to explore which promotion strategies should be used to attract especially older and lower educated people to the online Adult Health Monitor questionnaire. Furthermore, effort should be put into increasing interest in the computer tailored lifestyle intervention among online Adult Health Monitor participants and to persuade them to actually use the intervention.

### Strengths and limitations

One of the major strengths of this study was the opportunity to proactively offer an Internet-delivered computer tailored lifestyle intervention to a large sample of Dutch inhabitants within the controlled environment of the Adult Health Monitor. This allowed us to carefully determine the number of people invited and eligible for participation, as well the number of actual intervention participants. As a result, valuable information was obtained regarding actual numbers of reach of the used dissemination strategy. Furthermore, detailed information was gathered regarding not only characteristics of people that were interested and participated in the intervention, but also of people that refrained from participation. This is exceptional, since a problem encountered in most studies evaluating dissemination strategies is the accuracy by which all non-users can be tracked. Thus, the current study provided a unique possibility to obtain information on so-called non-users (e.g. people not interested in the intervention or people that were interested, but refrained from actual participation).

The study also suffered from several limitations. First, the sample used for this study was a convenience sample, consisting of people from two Dutch provinces. However, it is important to stress that all RPHS’s invite an initial sample which is representative of the Dutch population, to participate in the Adult Health Monitor [30]. However, this study might have been susceptible to selection bias. Within this study, we tried to optimize reach of a computer tailored lifestyle intervention by embedding the intervention in the Adult Health Monitor. Since this intervention was an Internet-delivered intervention it was only offered to people that decided to fill out the electronic version of the Adult Health Monitor. Results have indicated that these specific electronic participants tend to be younger, have a relatively higher education and an unhealthier lifestyle. As a result, data on actual intervention participation were obtained from a selective sample. This sample does not provide a good cross-section of the general Dutch population [61], which implies that the obtained results should be generalized with caution. Previous studies have, however, indicated that Internet-delivered computer tailored interventions tend to predominantly reach higher educated people [29,62], which might imply that our sample partly corresponds to the subgroup of people known to be reached by Internet-delivered computer tailored interventions. Although it does not represent the general population, the obtained results are still valuable in the context of Internet-delivered computer tailored lifestyle intervention.

## Conclusions

The present study used a proactive strategy to increase reach of an Internet-delivered multi component computer tailored intervention, by embedding the intervention in an existing online health monitoring system of the Regional Public Health Services in the Netherlands. With one out of three online Adult Health Monitor participants actually using the intervention, the employed proactive dissemination strategy succeeded in ensuring relatively high levels of reach. With regard to adequacy of reach result from this study indicated that reach among at-risk individuals (e.g. low socioeconomic status and unhealthy lifestyle) remained modest. We therefore recommend to further optimize reach by putting additional effort into increasing interest in the lifestyle intervention among at-risk individuals and to encourage them to actually use the intervention.

## Abbreviations

RPHS: Regional public health services; BMI: Body mass index; SQUASH: Short QUestionnaire to ASsess health enhancing physical activity; FFQ: Food frequency questionnaire; QFV: Quantity frequency variability questionnaire; K10: Kessler psychological distress scale; SF-12: Medical outcomes study 12-item short form health survey; SF-36: Medical outcomes study 36-item short form health survey.

## Competing interest

Hein de Vries is the scientific director of Vision2Health, a company that licenses evidence-based innovative computer-tailored health communication tools.

## Authors’ contributions

All authors substantially contributed to the development of the study, and interpretation of the data. FS drafted the manuscript and DS, LP, HdV and LvO substantially contributed to revising it. All authors approved the final version of the manuscript.

## Pre-publication history

The pre-publication history for this paper can be accessed here:

http://www.biomedcentral.com/1471-2458/13/721/prepub
